# Adaptive Response in Mice Exposed to 900 MHz Radiofrequency Fields: Primary DNA Damage

**DOI:** 10.1371/journal.pone.0032040

**Published:** 2012-02-28

**Authors:** Bingcheng Jiang, Jihua Nie, Zhen Zhou, Jie Zhang, Jian Tong, Yi Cao

**Affiliations:** School of Public Health, Soochow University, Suzhou, Jiangsu, People's Republic of China; University of Texas Health Science Center at San Antonio, United States of America

## Abstract

The phenomenon of adaptive response (AR) in animal and human cells exposed to ionizing radiation is well documented in scientific literature. We have examined whether such AR could be induced in mice exposed to non-ionizing radiofrequency fields (RF) used for wireless communications. Mice were pre-exposed to 900 MHz RF at 120 µW/cm^2^ power density for 4 hours/day for 1, 3, 5, 7 and 14 days and then subjected to an acute dose of 3 Gy γ-radiation. The primary DNA damage in the form of alkali labile base damage and single strand breaks in the DNA of peripheral blood leukocytes was determined using the alkaline comet assay. The results indicated that the extent of damage in mice which were pre-exposed to RF for 1 day and then subjected to γ-radiation was similar and not significantly different from those exposed to γ-radiation alone. However, mice which were pre-exposed to RF for 3, 5, 7 and 14 days showed progressively decreased damage and was significantly different from those exposed to γ-radiation alone. Thus, the data indicated that RF pre-exposure is capable of inducing AR and suggested that the pre-exposure for more than 4 hours for 1 day is necessary to elicit such AR.

## Introduction

Induction of adaptive response (AR) in mammalian cells exposed to ionizing radiation (IR) is well documented in scientific literature. Animal and human cells which were pre-exposed to a very low, non-genotoxic adaptive dose (AD) of IR were found to be resistant to the damage induced by subsequent exposure to a higher, genotoxic challenge dose (CD) of IR as well as chemical mutagens. Non-ionizing radiofrequency fields (RF) in the frequency range 800–2000 MHz are increasingly used in wireless communication systems in recent years and the conclusion from several reviewers was that RF exposure is non-genotoxic [1]–[9]. In our earlier studies, we have examined whether RF is capable of inducing AR to subsequent exposure with IR [10], [11]. Mice were pre-exposed to 900 MHz RF at 12, 120 and 1200 µW/cm^2^ power density (548 mW/kg specific absorption rate) for 1 hour/day for 14 days (AD) and then subjected to a high dose of γ-radiation (CD). The results indicated that the animals which were exposed to AD+CD showed significantly reduced hematopoietic tissue damage assessed from colony forming cells in the bone marrow and spleen and, increased survival as compared to those exposed to CD alone. Also, the response (AR) was maximal when the mice were pre-exposed to RF at 120 µW/cm^2^ power density. In the present study, we have evaluated the extent of primary DNA damage in the form of alkali base damage and single strand breaks in the DNA in peripheral blood leukocyte in mice pre-exposed to 900 MHz RF at 120 µW/cm^2^ power density for 4 hours/day for 1, 3, 5, 7 and 14 days (AD) and then subjected to 3 Gy γ-radiation (CD). A significant reduction in damage was observed in mice exposed to AD+CD as compared to those subjected CD alone suggesting that RF pre-exposure was capable of inducing AR and more than 4 hours for 1 day exposure is necessary to elicit such response.

## Materials and Methods

### Ethics Statement

All animal handling procedures were reviewed and approved by the Animal Care/User Ethical Committee of Soochow University, Suzhou City, P.R.China (approval number A68–2011).

### Animal Procurement and Handling

Adult male ICR mice weighing approximately 25 gm were obtained from the Animal Center, Suzhou University and housed in a facility maintaining 22+/−1°C temperature, 50+/−5% relative humidity and 12-hour light-dark cycles. All mice were fed on commercial diet (Suzhou Shuangshi Laboratory Animal Feed Science Co. Ltd., Suzhou City, Jiangsu Province, China) and provided water *ad libitum*. After 7 days quarantine period, the animals were randomly divided into several groups of 5 mice each: (a) un-exposed controls, (b) acute exposure to 3 Gy ^60^Co γ-radiation (Nordion, Ottawa, ON, Canada, dose rate 0.5 Gy/minute), (c) 900 MHz RF at 120 µW/cm^2^ power density for 4 hours/day for 1, 3, 5, 7 and 14 days, and (d) the same RF exposure periods+3 Gy ^60^Co γ-radiation given at 4 hour after RF exposure on days 1, 3, 5, 7 and 14.

### RF Exposure

Small plastic boxes, designed in-house to hold a single restrained mouse, were used for RF exposure. The RF exposure system was described in detail in our earlier paper [11]. Briefly, it consisted of a signal generator (SN2130J6030, Narda STS S.r.l., Italy), power amplifier (SN1020, HD communications Corp, USA) and GTEM chamber (Giga-hertz Transverse ElectroMagnetic chamber, built in-house at Suzhou University, China). The signal generated was amplified and transmitted to GTEM chamber. The 120 µW/cm^2^ power density was controlled by a computer which also monitored and recorded in a logging system. The specific absorption rate (SAR), 548 mW/kg, was calculated using finite-difference-time-domain (FDTD) model [11]. The environmental conditions during and after RF exposures were kept identical through out the study period.

### Blood Collection and Comet Assay

The interval between RF pre-exposure (1, 3, 5, 7 and 14 days) and γ-radiation was 4 hours. Then, the animals were anesthetized using carbon-di-oxide and 300–500 µl of peripheral blood was collected by heart puncture. Leukocytes were isolated immediately using Histopaque 1083 (Sigma-Aldrich, USA), washed with phosphate buffered saline and diluted to obtain 10^5^ cells/ml. The alkaline comet assay described by Singh et al [12] was followed with minor modifications. The cells were lysed (pH 10), unwind the DNA in alkaline buffer (pH 13) for 30 minutes followed by electrophoresis for 30 min (20 V, 300 mA). After neutralization (pH 7.5) the cells were stained with ethidium bromide (20 µg/ml). All slides were coded before microscopic examination. A fluorescence microscope equipped with CASP software (CASP Lab, Poland) was used to record comet tail length (TL) and tail moment (TM). For each animal and for each exposure, 50 comets were analyzed.

### Statistical Analysis

For each RF exposure duration the data obtained in different groups of mice for TL and TM was subjected to separate statistical analyses using software version 8.2. One-way analysis of variance (ANOVA), Student's *t-test* with pair wise comparisons were used to obtain significant differences, if any, between different groups of mice for each exposure duration. A *p* value<0.05 was considered as significant difference between groups.

## Results

There were no significant differences in response among the 5 mice in all groups exposed to RF alone (AD), γ-radiation alone (CD) and to the combined exposures (AD+CD). Hence, the results were pooled and the mean+/−standard deviations are presented in Table 1. The tail length (TL) and tail moment (TM) in leukocytes of control animals and those that were pre-exposed to RF alone for 4 hours/day for 1, 3, 5, 7 and 14 days (AD) were similar and no significant differences were observed. Acute exposure to 3 Gy γ-radiation alone (CD) resulted in significant increases in TL and TM (*p* = 0.01).

**Table 1:** Primary DNA damage, comet tail length and tail moment, in peripheral blood leukocytes of mice in different experimental groups.

Group	RF exposure				
	1 days	3 days	5 days	7 days	14 days
Tail Length (TL)					
(1) Controls (no RF)	3.3±0.15	3.1±0.06	3.2±0.10	3.0±0.17	3.1±0.07
(2) GR (3 Gy)*	12.8±1.28	12.6±1.46	12.5±1.26	12.3±1.22	12.4±1.25
(3) 900 MHz RF	4.2±0.39	3.4±0.13	3.3±0.11	3.2±0.08	3.2±0.09
(4) 900 MHz RF+GR	11.1±0.87	6.5±0.35	4.0±0.47	3.3±0.15	4.2±0.28
Expected values**	13.7	12.9	12.6	12.5	12.5
% Decrease	19	50	68	74	66
Group 1 versus 2	*p* = 0.000	*p* = 0.000	*p* = 0.000	*p* = 0.000	*p* = 0.000
Group 1 versus 3	*p* = 0.441	*p* = 0.650	*p* = 0.931	*p* = 0.788	*p* = 0.881
Group 2 versus 4	*p* = 0.153	*p* = 0.000	*p* = 0.000	*p* = 0.000	*p* = 0.000
Tail Moment (TM)					
(1) Controls (no RF)	0.1±0.01	0.1±0.01	0.1±0.01	0.0±0.01	0.1±0.02
(2) GR (3 Gy)*	1.3±0.17	1.5±0.24	1.4±0.22	1.4±0.22	1.4±0.22
(3) 900 MHz RF	0.2±0.04	0.2±0.03	0.2±0.02	0.1±0.02	0.1±0.01
(4) 900 MHz RF+GR	1.1±0.14	0.6±0.04	0.2±0.07	0.1±0.02	0.2±0.03
Expected values**	1.4	1.6	1.5	1.5	1.4
% Decrease	21	63	87	93	86
Group 1 versus 2	*p* = 0.000	*p* = 0.000	*p* = 0.000	*p* = 0.000	*p* = 0.000
Group 1 versus 3	*p* = 0.755	*p* = 0.672	*p* = 0.758	*p* = 0.859	*p* = 0.976
Group 2 versus 4	*p* = 0.196	*p* = 0.000	*p* = 0.000	*p* = 0.000	*p* = 0.000

Number of animals in each group - 5. Data are Mean+/−Standard Deviation.

*No RF exposure. 3 Gy γ-radiation (GR) exposure is acute on days 1, 3, 5, 7 and 14 days.

**Expected values are the sum of two individual treatments (GR alone+RF alone) minus controls.

The TL and TM in mice which were pre-exposed to RF for 4 hours for 1 day (AD) and then subjected to γ-irradiation (CD) showed similar extent of damage and not significantly different from those exposed to CD alone. However, mice which were pre-exposed to AD for 3, 5, 7 and 14 days and then subjected to CD showed significantly decreased TL and TM as compared to those exposed to CD alone (p<0.01). The extent of decrease was correlated with the number of RF pre-exposures and maximal after 7 days pre-exposure (19% to 74% for TL and from 21% to 93% for TM). The % decrease in TL and TM observed after 14 days pre-exposure was smaller than that after 7 days but still, it was significantly different from the mice exposed to CD alone.

## Discussion

The alkaline comet assay is considered as sensitive and simple method to detect the primary DNA damage (alkali-labile lesions and single-strand breaks) in individual cells. This method was used to examine whether pre-exposure of mice to 900 MHz RF at 120 µW/cm^2^ power density (AD) is able to induce AR to subsequent exposure to γ-radiation (CD). The results indicated similar extent of damage between control mice and those pre-exposed to RF (AD) *per se* while a significant increase in damage was observed in animals subjected to CD alone. However, compared with mice exposed to CD alone, the damage was significantly reduced in mice which were pre-exposed AD for 4 hours/day for 3, 5, 7 and 14 days and then subjected to CD. However, such decrease was not observed after 1 day pre-exposure to AD. Thus, the results suggested that RF pre-exposure is capable of inducing AR and that more than 4 hours (1 day) pre-exposure is necessary for the induction of such AR.

Induction of primary DNA damage following RF exposure *per se* is controversial in animal investigations. Lai and Singh [13], [14] reported a significant increase in single- and double-strand breaks in the DNA of rat brain cells immediately and at 4 hours after exposure to 2450 MHz RF for 2 hours. Such observations were not confirmed in subsequent investigations [15]. The results from this study indicated no significant difference in damage between control mice and those exposed to RF alone. However, 900 MHz RF and mouse leukocytes were used in the current study where as 2450 MHz RF and rat brain cells were used in the above mentioned reports.

Induction of AR in animals exposed to IR (AD+CD) has been reported [16]. With respect to RF-induced AR to subsequent IR exposure, Mortazavi et al [17] exposed Sprague Dawley rats to 900 MHz RF at 2 W power density (AD) for a total of 6 hours/day (3 h on/12 h off cycles for 4 days) for 4 days and then subjected the animals to whole-body LD_50/30_ dose of 8 Gy γ-radiation (CD). A significant increase in survival was observed in mice exposed to AD+CD compared to those subjected to CD alone. These results confirmed our earlier observations of increased survival and reduced hematopoietic tissue damage in mice which were pre-exposed to RF [11] and also gives support to the current observations made at a much lower 120 µW/cm^2^ power density. To the best of our knowledge, there were, at least, two reports of RF-induced AR in human blood lymphocytes. Sannino et al [18], [19] stimulated the cells with phytohemagglutinin for 24 hours and then exposed to 900 MHz for 20 hours (AD) followed by a challenge dose (CD) with the genotoxic chemical mutagen, mitomycin C (MMC). In both reports, the incidence of micronuclei was significantly lower in lymphocytes exposed to AD+CD compared to those exposed to CD alone. These data are important and suggested that RF pre-exposure can induce resistance to subsequent genetic damage induced not only by IR (as in the current study) but also by a chemical mutagen (MMC). The primary damage assessed in the current study is the first step in the induction of genetic damage, and un-/mis-repair of such damage would result in the formation of chromosomal aberrations and micronuclei. The significantly reduced primary damage observed in the present study in mice exposed to AD+CD would lend support to the decreased micronuclei reported by Sannino et al [18], [19]. Thus, the overall data indicated that RF is capable of inducing AR in rodents and in human cells, and in mice, RF pre-exposure for more than 4 hours (for 1 day) is necessary to elicit AR.

Several investigators have reported inter-individual variation in RF- and IR-induced AR [18], [19], [20]–[22] in human blood lymphocytes. The observations made in this study did not indicate significant differences between the mice studied. The small sample size of 5 mice in each group might not have been enough to derive such conclusion.

The mechanism by which RF is able to induce AR is not fully investigated. Perhaps, RF exposure given as AD produces a ‘signal or trigger’ that does not induce significant genetic damage but offers protection to subsequent damage induced by genotoxic CD. Several other hypotheses have been proposed in the case of IR-induced AR: these include and not limited to efficient repair of damaged DNA, potential role for DNA repair enzymes and protein synthesis [23]–[28]. In this study, we have observed a significant reduction in primary damage in mice exposed to AD+CD. It is still to be determined if the primary damage can be repaired efficiently and at a faster rate.
